# Supplementary material for: To what extent does self-reported physical activity impact children’s wellbeing and mental health? Insights from school-aged children in Wales
Source: PLoS One. 2025 May 22;20(5):e0313970. doi: 10.1371/journal.pone.0313970 (PMC12097639; doi:10.1371/journal.pone.0313970)
Supplement: S1 File — This is a copy of the HAPPEN Survey from 2023–2024. (PDF) [file pone.0313970.s001.pdf]

The HAPPEN Survey 2023/2024

This survey is going to ask you all about your health.

Click start to begin.

* Required

Consent Form

Before you start please click this link to read the information sheet -> https://happen-wales.co.uk/childrens-information-sheet/

1

I have read the child information sheet -> https://happen-wales.co.uk/childrens-information-sheet/ (click the link if you haven't read it) and understand that if I take part I can change my mind at any time, and this will not be a problem at all. *

- Yes
- No

2

I am happy for you to use my questionnaire for research. Only the researchers in the team will know my name and will not tell anyone else my answers. *

- Yes
- No do not use my questionnaire

3

I am happy for you to look at my school and health records to see how my school is doing (as a group). This is anonymous which means I cannot be identified. *

- Yes
- No

About You

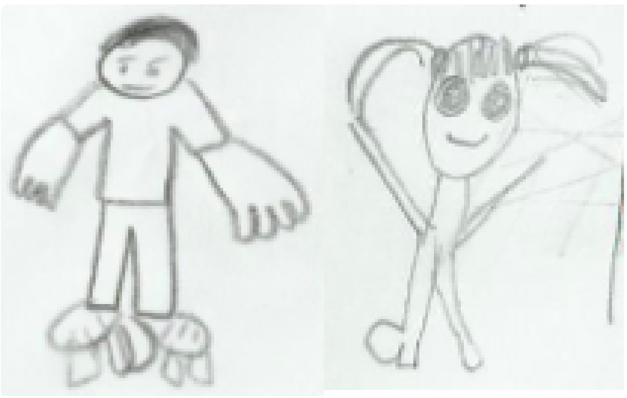

4

First Name

5

Last Name

6

Home Post Code

7

What school do you go to?

8

Do you have any other children living in your house with you?

9

What year are you in?

- Year 3
- Year 4
- Year 5
- Year 6

10

Do you have a garden?

- ◯ Yes
- ◯ No

11

Gender

- ◯ Boy
- ◯ Girl
- ◯ Prefer not to say

12

Are you...

- ◯ Asian
- ◯ Black
- ◯ White
- ◯ Mixed
- ◯ Prefer not to say

13

Date of Birth: Year

- ◯ 2010
- ◯ 2011
- ◯ 2012
- ◯ 2013
- ◯ 2014
- ◯ 2015

14

Date of Birth: Month

- January
- February
- March
- April
- May
- June
- July
- August
- September
- October
- November
- December

15

Date of Birth: Day

- ○ 1
- ○ 2
- ○ 3
- ○ 4
- ○ 5
- ○ 6
- ○ 7
- ○ 8
- ○ 9
- ○ 10
- ○ 11
- ○ 12
- ○ 13
- ○ 14
- ○ 15
- ○ 16
- ○ 17
- ○ 18
- ○ 19

- ○ [illegible]
- ○ 20
- ○ 21
- ○ 22
- ○ 23
- ○ 24
- ○ 25
- ○ 26
- ○ 27
- ○ 28
- ○ 29
- ○ 30
- ○ 31

Yesterday

Firstly, think carefully about what you did <u>YESTERDAY</u> and then answer the following questions....

16

Did you have breakfast yesterday?

- ◯ Yes - At Home
- ◯ Yes - At School
- ◯ No

17

How did you get to school YESTERDAY?

- On the bus
- On bike
- In the car/taxi
- Walked
- Ran/jogged
- Scooter
- Skateboarded/Rollerbladed

18

What did you have to eat for lunch YESTERDAY?

- School dinner
- Packed lunch from home
- Nothing

19

What did you do for MOST of your break-times YESTERDAY? (This includes lunchtime)

- ◯ Sat around inside or outside
- ◯ Ran around
- ◯ Stood around
- ◯ Walked around

20

How many friends did you play with?

- ◯ I like to play on my own
- ◯ 1-2
- ◯ 3-4
- ◯ 5 or more

21

Did you have an afternoon break at school?

- ◯ Yes
- ◯ No

22

How did you get home YESTERDAY?

- On the bus
- On bike
- In the car/taxi
- Walked
- Ran/jogged
- Scooter
- Skateboarded/Rollerbladed

After School

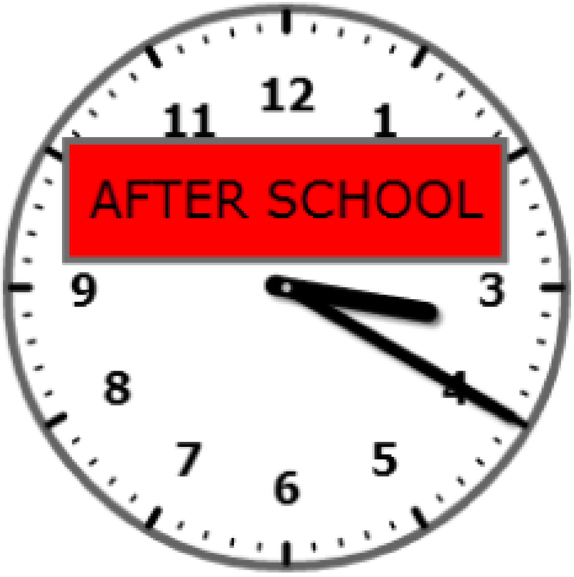

23

How many portions of fruit and vegetables did you eat YESTERDAY?

- 1
- 2
- 3
- 4
- 5
- 6
- 7
- 8

24

How many times did you brush your teeth YESTERDAY?

- 0
- 1
- 2
- 3

25

What time did you fall asleep YESTERDAY (to the nearest half hour)?

- 7:00pm
- 7:30pm
- 8:00pm
- 8:30pm
- 9:00pm
- 9:30pm
- 10:00pm
- 10:30pm
- 11:00pm
- 11:30pm
- 12:00am
- 12:30am
- 1:00am
- 1:30am
- 2:00am
- 2:30am
- 3:00am
- 3:30am
- 4:00am

4:00am

26

What time did you wake up TODAY (to the nearest half hour)?

- 5:00am
- 5:30am
- 6:00am
- 6:30am
- 7:00am
- 7:30am
- 8:00am
- 8:30am
- 9:00am

THE LAST WEEK

NOW think about what you did in the last 7 days...

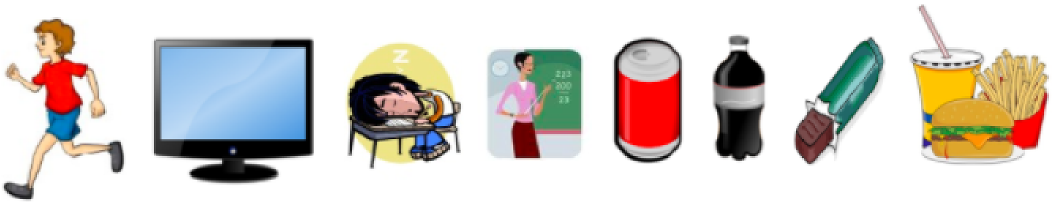

27

In the last 7 days, how many days did you do sports or exercise for at least 1 hour in total (This includes doing any activities or playing sports where your heart beat faster, you breathed faster and you felt warmer?

- ◯ 0 days
- ◯ 1-2 days
- ◯ 3-4 days
- ◯ 5-6 days
- ◯ 7 days

28

In the last 7 days, how many days did you watch TV/play online games/use the internet etc. for 2 or more hours a day (in total)?

- 0 days
- 1-2 days
- 3-4 days
- 5-6 days
- 7 days

29

In the last 7 days, how many days did you feel tired?

- 0 days
- 1-2 days
- 3-4 days
- 5-6 days
- 7 days

30

In the last 7 days, how many days did you feel like you could concentrate/pay attention well in class?

- 0 days
- 1-2 days
- 3-4 days
- 5-6 days
- 7 days

31

In the last 7 days, how many days did you drink at least one fizzy drink (e.g. coke, fanta, sprite)

- 0 days
- 1-2 days
- 3-4 days
- 5-6 days
- 7 days

32

In the last 7 days, how many days did you eat at least one sugary snack (e.g. chocolate bar, sweets)

- 0 days
- 1-2 days
- 3-4 days
- 5-6 days
- 7 days

33

In the last 7 days, how many days did you eat take away foods (e.g. McDonalds, KFC, chinese)

- 0 days
- 1-2 days
- 3-4 days
- 5-6 days
- 7 days

Sport and Activity

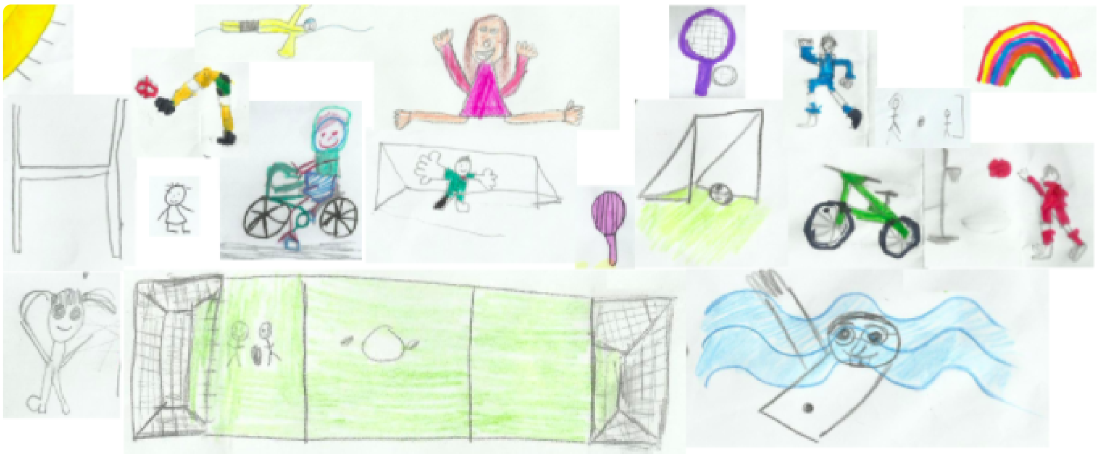

34

These questions are going to ask you how you feel about physical activity (This includes any activity where your heart beats faster, you breathe faster and you feel warmer)

	Strongly agree	Agree	Disagree	Strongly disagree
I want to take part in physical activity	◯	◯	◯	◯
I feel confident to take part in lots of different physical activities	◯	◯	◯	◯
I am good at lots of different physical activities	◯	◯	◯	◯
I understand why taking part in physical activity is good for me	◯	◯	◯	◯

35

What motivates you the most to take part in physical activity and sport?

	Strongly agree	Agree	Disagree	Strongly disagree
I am driven by enjoyment	○	○	○	○
I am driven by having fun with my friends	○	○	○	○
I am driven by learning and improving my skills	○	○	○	○
I am driven by competing against myself	○	○	○	○
I am driven by playing and competing in a team	○	○	○	○

36

Is anyone else at home physically active/do they play a sport?

○ Yes

○ No

37

If yes, who is this?

- Mum
- Dad
- Brother
- Sister
- Grandparents
- Aunty
- Uncle

38

How many times do you take part in a sports club OUTSIDE OF SCHOOL each week?

- 0
- 1
- 2
- 3
- 4
- 5
- 6
- 7
- 8
- 9
- 10

39

Can you ride a bike WITHOUT STABILISERS?

- Yes
- No

40

Can you swim 25 metres WITHOUT A FLOAT OR ARMBANDS? (This is 1 length of a standard swimming pool)

- Yes
- No

You and Your Feelings

This part of the survey is going to ask you how you feel. There are no right or wrong answers. You should just pick the answer which is best for you.

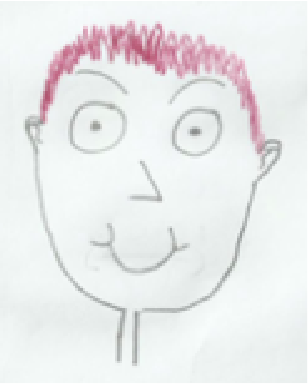
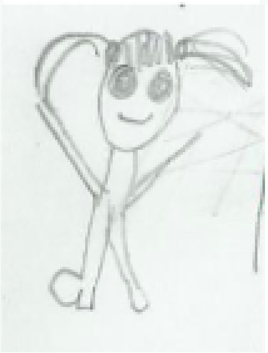
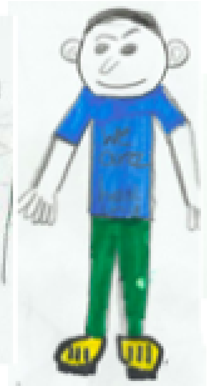
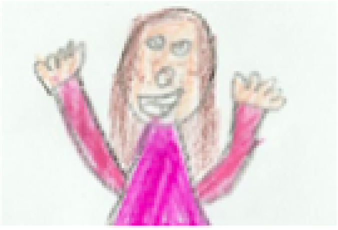

41

Tell us if you agree or disagree with the following:

	Strongly agree	Agree	Don't agree or disagree	Disagree	Strongly disagree
I am doing well at school	○	○	○	○	○
I feel part of my school community	○	○	○	○	○
I have lots of choice over things that are important to me	○	○	○	○	○
There are lots of things I'm good at	○	○	○	○	○
I have someone to talk to at home	○	○	○	○	○
I feel safe in school	○	○	○	○	○
I have someone to talk to in school	○	○	○	○	○

42

On a scale of 1 to 10 (1 being very unhappy and 10 being very happy, how do you feel about:

Your Health

☆ ☆ ☆ ☆ ☆ ☆ ☆ ☆ ☆ ☆

43

On a scale of 1 to 10 (1 being very unhappy and 10 being very happy, how do you feel about:

Your School

☆ ☆ ☆ ☆ ☆ ☆ ☆ ☆ ☆ ☆

44

On a scale of 1 to 10 (1 being very unhappy and 10 being very happy, how do you feel about:

Your Family

☆ ☆ ☆ ☆ ☆ ☆ ☆ ☆ ☆ ☆

45

On a scale of 1 to 10 (1 being very unhappy and 10 being very happy, how do you feel about:

Your Friends

☆ ☆ ☆ ☆ ☆ ☆ ☆ ☆ ☆ ☆

46

On a scale of 1 to 10 (1 being very unhappy and 10 being very happy, how do you feel about:

Your Appearance (how you look)

☆ ☆ ☆ ☆ ☆ ☆ ☆ ☆ ☆ ☆

47

On a scale of 1 to 10 (1 being very unhappy and 10 being very happy, how do you feel about:

Your Life

☆ ☆ ☆ ☆ ☆ ☆ ☆ ☆ ☆ ☆

You and Your Feelings

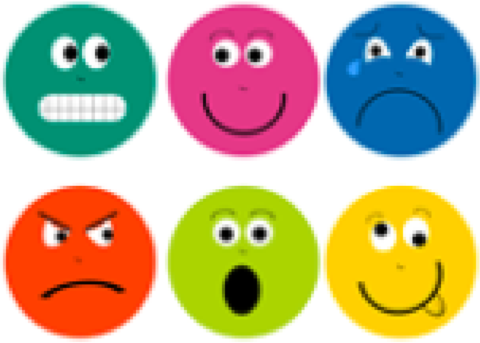

Based on the Me and My Feelings Questionnaire (Deighton, Tymms, Vostanis, Belsky, Fonagy, Brown, Martin, Patalay, & Wolpert, 2012)

48

Remember, there are no right or wrong answers, just pick which is right for you.

	Never	Sometimes	Always
I feel lonely	○	○	○
I cry a lot	○	○	○
I am unhappy	○	○	○
I feel nobody likes me	○	○	○
I worry a lot	○	○	○
I have problems sleeping	○	○	○
I wake up in the night	○	○	○
I am shy	○	○	○
I feel scared	○	○	○
I worry when I am at school	○	○	○
I get very angry	○	○	○
I lose my temper	○	○	○
I hit out when I am angry	○	○	○
I do things to hurt people	○	○	○
I am calm	○	○	○

I break things on purpose	◯	◯	◯

Your Local Area

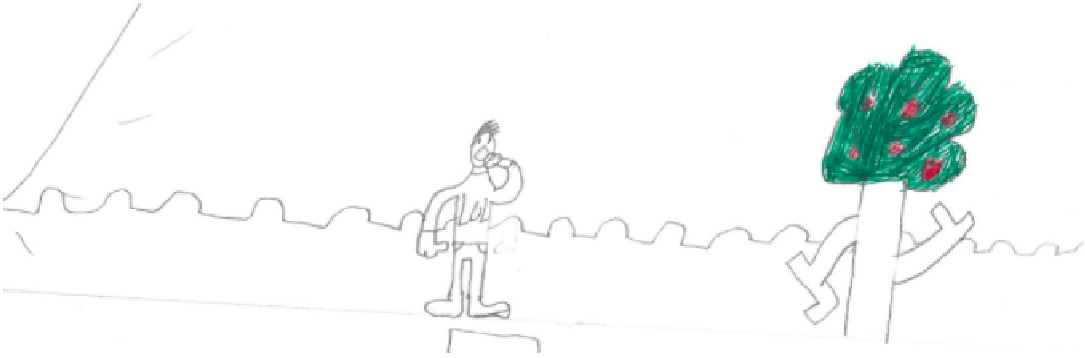

49

On a scale of 1 to 10 (1 being not very safe and 10 being very safe), how safe do you feel playing in your area?

☆ ☆ ☆ ☆ ☆ ☆ ☆ ☆ ☆ ☆

50

From your house, can you walk to school?

- ◯ Yes
- ◯ No

51

From your house, can you easily walk to a park?

- Yes
- No

52

From your house, can you easily walk to a leisure centre/sports centre?

- Yes
- No

53

Can you play in all the places you would like to?

- I can play in all the places I would like to
- I can play in some of the places I would like to
- I can only play in a few places I would like to
- I can hardly play in any of the places I would like to

54

Are you happy with the area that you live in?

- Yes
- No

55

If you could change something to make you and your friends healthier and happier, what would you change... IN SCHOOL?

56

If you could change something to make you and your friends healthier and happier, what would you change... OUT OF SCHOOL?

Don't forget to press submit below!

We have some resources on our website if you would like to learn more or would like to speak to someone... https://happen-wales.co.uk/some-resources-for-you/

This content is neither created nor endorsed by Microsoft. The data you submit will be sent to the form owner.

Microsoft Forms
