# Supplementary material for: To what extent does self-reported physical activity impact children’s wellbeing and mental health? Insights from school-aged children in Wales
Source: PLoS One. 2025 May 22;20(5):e0313970. doi: 10.1371/journal.pone.0313970 (PMC12097639; doi:10.1371/journal.pone.0313970)
Supplement: S2 File — Outlining the variables that were used in the analysis of this study. (PDF) [file pone.0313970.s002.pdf]

HAPPEN
VARIABLES IN ANALYSIS

Variable Name	Data	Date Type	Notes
Gender	Self-reported gender of participant	Continuous	1=Boy<br>2=Girl<br>9=Prefer not to say
WIMD2019Quintile	Deprivation based on WIMD Index 2019	Quintile	1=Most deprived<br>5=Least deprived
Overall Wellbeing Score	Wellbeing based on GCI	Continuous	0=Low Wellbeing<br>5=High Wellbeing
GCI: ChildWBHealth	*How happy are you with your health?*	*Continuous*	*0=Very unhappy*<br>*10=Very happy*
GCI: ChildWBFitness	*How do you feel about your fitness?*	*Continuous*	*0=Very unhappy*<br>*10=Very happy*
GCI: ChildWBSchool	*How happy are you with your school?*	*Continuous*	*0=Very unhappy*<br>*10=Very happy*
GCI: ChildWBFamily	*How happy are you with your family?*	*Continuous*	*0=Very unhappy*<br>*10=Very happy*
GCI: ChildWBFriends	*How happy are you with your friends?*	*Continuous*	*0=Very unhappy*<br>*10=Very happy*
Overall Physical Literacy Score	Physical literacy score based on PL questions	Continuous	0=Low PL<br>4=High PL
physlittakepart	*I want to take part in activity*	*Scale*	*1=Strongly disagree*<br>*2=Disagree*<br>*3=Agree*<br>*4=Strongly agree*
physlitfeelconfident	*I feel confident to take part*	*Scale*	*1=Strongly disagree*<br>*2=Disagree*<br>*3=Agree*<br>*4=Strongly agree*
physlitgood	*I am good at lots of activities*	*Scale*	*1=Strongly disagree*<br>*2=Disagree*<br>*3=Agree*<br>*4=Strongly agree*
physlitunderstand	*I understand why activity is good for me*	*Scale*	*1=Strongly disagree*<br>*2=Disagree*<br>*3=Agree*<br>*4=Strongly agree*
Ability to Swim 25m	Are you able to swim 25m?	Binary	0 = No<br>1 = Yes

Ability to Ride A Bike	Are you able to ride a bike?	Binary	0 = No<br>1 = Yes
Active Travel To School	Self-reported active travel to school	Binary	0=No<br>1=Yes
Active Travel From School	Self-reported active travel from school	Binary	0=No<br>1=Yes
Safe in Area	On a scale of 1 – 10 how safe do you feel in your area	Continuous	0=Very unhappy<br>10=Very happy
Feel Autonomous	I have lots of choice over things that are important to me?	Scale	1 = Strongly disagree<br>2 = Disagree<br>3 = Don't agree or disagee<br>4 = Agree<br>5 = Strongly agree
Feel Competent	I am doing well	Scale	1=Strongly disagree<br>2=Disagree<br>3=Don't agree or disagee<br>4=Agree<br>5=Strongly agree
Emotional Difficulties	Continuous score for emotional difficulty based off Me and My Feelings	Continuous	>12 Clinically Significant
Behavioural Difficulties	Continuous score for behavioural difficulty based off Me and My Feelings	Continuous	>7 Clinically Significant
Me and My Feelings Questionnaire (Deighton et al. 2012):<br><br>*Mhfeellonely (I feel lonely)*<br>*Mhcrylot (I cry a lot)*<br>*Mhunhappy (I am unhappy)*<br>*Mhnobodylikesme (I feel nobody likes me)*<br>*Mhworrylot (I worry a lot)*<br>*Mhproblemsleeping (I have problems sleeping)*<br>*Mhwakeupinnight (I wake up in the night)*<br>*Mhshy (I am shy)*<br>*Mhfeelscared (I feel scared)*<br>*Mhworryatschool (I worry when I am at school)*<br>*Mhgetveryangry (I get very angry)*	*Measures emotional/behavioural difficulties*	*Scale*	*0 = Never*<br>*1 = Sometimes*<br>*2 = Never*

Mhlosetemper (I lose my temper)<br>*Mhhitout (I hit out when I am angry)*<br>*Mhdothingstohurt (I do things to hurt people)*<br>*Mhcalm (I am calm)*<br>*Mhbreakthings (I break things on purpose)*			
